# Characterization of singlet oxygen-accumulating mutants isolated in a screen for altered oxidative stress response in *Chlamydomonas reinhardtii*

**DOI:** 10.1186/1471-2229-10-279

**Published:** 2010-12-17

**Authors:** Beat B Fischer, Rik IL Eggen, Krishna K Niyogi

**Affiliations:** 1 Department of Plant and Microbial Biology, University of California, Berkeley, CA 94720-3102 USA; 2 Eawag, Swiss Federal Institute of Aquatic Science and Technology, Department of Environmental Toxicology, Ueberlandstrasse 133, CH-8600 Dübendorf, Switzerland

## Abstract

**Background:**

When photosynthetic organisms are exposed to harsh environmental conditions such as high light intensities or cold stress, the production of reactive oxygen species like singlet oxygen is stimulated in the chloroplast. In *Chlamydomonas reinhardtii *singlet oxygen was shown to act as a specific signal inducing the expression of the nuclear glutathione peroxidase gene *GPXH/GPX5 *during high light stress, but little is known about the cellular mechanisms involved in this response. To investigate components affecting singlet oxygen signaling in *C. reinhardtii*, a mutant screen was performed.

**Results:**

Mutants with altered *GPXH *response were isolated from UV-mutagenized cells containing a *GPXH*-arylsulfatase reporter gene construct. Out of 5500 clones tested, no mutant deficient in *GPXH *induction was isolated, whereas several clones showed constitutive high *GPXH *expression under normal light conditions. Many of these *GPXH *overexpressor (*gox*) mutants exhibited higher resistance to oxidative stress conditions whereas others were sensitive to high light intensities. Interestingly, most *gox *mutants produced increased singlet oxygen levels correlating with high *GPXH *expression. Furthermore, different patterns of altered photoprotective parameters like non-photochemical quenching, carotenoid contents and α-tocopherol levels were detected in the various *gox *mutants.

**Conclusions:**

Screening for mutants with altered *GPXH *expression resulted in the isolation of many *gox *mutants with increased singlet oxygen production, showing the relevance of controlling the production of this ROS in photosynthetic organisms. Phenotypic characterization of these *gox *mutants indicated that the mutations might lead to either stimulated triplet chlorophyll and singlet oxygen formation or reduced detoxification of singlet oxygen in the chloroplast. Furthermore, changes in multiple protection mechanisms might be responsible for high singlet oxygen formation and *GPXH *expression, which could either result from mutations in multiple loci or in a single gene encoding for a global regulator of cellular photoprotection mechanisms.

## Background

Light energy is essential for growth of photosynthetic organisms but it can also harm them. Excess light can lead to the increased production of reactive oxygen species (ROS) which can damage cellular components such as lipids, proteins and DNA. Mainly at photosystem (PS) I but also at PSII, electron transfer reactions to molecular oxygen causes the production of superoxide anion radicals (O_2_^.-^), hydrogen peroxide (H_2_O_2_) and hydroxyl radicals (OH^.^) [1,2]. At PSII, triplet chlorophyll formation and the interaction with molecular oxygen stimulates the formation of singlet oxygen (^1^O_2_) [3]. Singlet oxygen was shown to contribute significantly to the ROS-induced cellular damage during high light stress [4] and consequently plant and algae have evolved efficient protection mechanisms to prevent the formation of this ROS. Some of these protection mechanisms can be detected as non-photochemical quenching (NPQ) of maximal chlorophyll fluorescence [5]. Short and long term acclimation processes like state transition (qT) or adjustment of PS stoichiometry help to prevent overreduction of the photosynthetic electron transport chain [6]. The energy-dependent quenching (qE) of excess light involves a ΔpH-induced activation of the xanthophyll cycle in which a violaxanthin de-epoxidase converts violaxanthin (V) into antheraxanthin (A) and zeaxanthin (Z) [7]. Increased levels of these xanthophylls together with the protonation of specific pigment-binding antenna proteins cause a conformational change of PSII into a high quenching state where excess light energy is dissipated as heat [5]. Additionally, zeaxanthin is an efficient ^1^O_2 _quencher and increased levels of this xanthophyll after exposure to high light conditions might reduce damage to membrane lipids [8,9]. Recently, two LHCSR3 genes have been found to be involved in NPQ in *Chlamydomonas reinhardtii *indicating that other unidentified components might function in photoprotection and prevention of ^1^O_2 _formation in photosynthetic organisms [10].

Singlet oxygen can damage the cell but it has also been found to play an important role in retrograde signaling through the specific activation of nuclear genes by plastid signals. Singlet oxygen produced in the chloroplast of the conditional *fluorescent *(*flu*) mutant was shown to stimulate the expression of a set of genes which was different from H_2_O_2 _induced genes [11]. Furthermore, O_2_^.-^/H_2_O_2 _exhibited an antagonizing effect on ^1^O_2_-induced gene expression in *flu *[12]. In a suppressor screen for the ^1^O_2_-induced programmed cell death response in *flu *mutants, two thylakoid-localized proteins, EXECUTER1 (EX1) and EXECUTER2 (EX2), were identified which are involved in the regulation of the ^1^O_2_-mediated genetic response [13,14]. In *C. reinhardtii*, the response to ^1^O_2 _has been studied using either specific exogenous photosensitizers like rose bengal (RB) or neutral red (NR) [15] or in strains lacking some ^1^O_2 _protective mechanisms like the xanthophyll-deficient mutant *npq1 lor1 *[16]. As found in *A. thaliana*, the response of the Chlamydomonas *HSP70A *gene to ^1^O_2 _could be distinguished from the response to H_2_O_2 _by different reporter constructs and was attributed to separate promoter regions [17]. Furthermore, the glutathione peroxidase homologous gene *GPXH/GPX5 *of *C. reinhardtii *was strongly induced by ^1^O_2 _but to a much lower extent by other ROS [18]. During high light stress, *GPXH *expression is strongly induced by ^1^O_2 _by transcriptional activation [19,20] and various regulatory elements in the promoter were required for induction by ^1^O_2 _[21]. The GPXH protein is predicted to be dual-targeted to the cytoplasm and the chloroplast, and its peroxidase activity with plastidial thioredoxin indicates a role in oxidative stress response of the chloroplast [21].

Even though ^1^O_2 _can function as a signal to activate nuclear gene expression, our knowledge of how the formation of ^1^O_2 _is controlled in photosynthetic organisms and which components are involved in the signal transduction from the plastid to the nucleus is still far from complete. Membrane lipids are primary targets of ^1^O_2_, and oxidized fatty acids could function as signaling intermediates [22,23]. However, experiments with carotenoid-depleted cultures indicated that in *C. reinhardtii *the sensor for ^1^O_2 _is not a lipophilic compound in the thylakoid membrane but probably is located in the aqueous phase of the chloroplast [24]. In an effort to identify components that affect the ^1^O_2 _induced genetic response in *C. reinhardtii*, we performed a mutant screen using a ^1^O_2_-specific *GPXH *reporter construct. Mutants with altered *GPXH *expression were isolated and characterized genetically and physiologically.

## Results

### Isolation of mutants with altered *GPXH *expression

**Figure 1:**
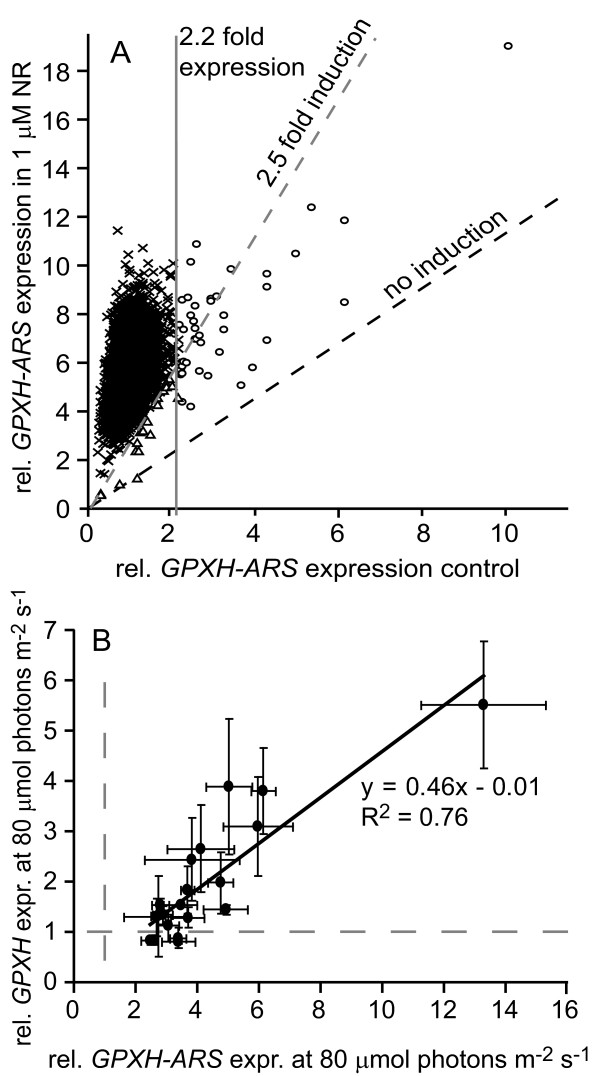
***GPXH-ARS *expression in the screened mutants**. **A**. 5500 clones were analyzed for the expression of the *GPXH*-*ARS *reporter construct under control or 2 μM NR-treated conditions. Clones with reduced induction by NR (< 2.5 fold, triangles) or increased basal expression under control condition (> 2.2 fold expression, circles) were selected for further analysis. **B**. Correlation of *GPXH *expression (wild-type gene) with the expression of the *GPXH*-*ARS *reporter construct in 20 *GPXH *overexpression (*gox*) mutants. Average expression was calculated from three independent experiments (± SE) and normalized to wild-type levels (grey dashed lines).

The expression of the *GPXH *gene is strongly induced by the increased production of ^1^O_2 _in the chloroplast. To identify components affecting ^1^O_2_-induced gene expression in *C. reinhardtii*, a mutant screen was performed using the *GPXH*-arylsulfatase (*GPXH-ARS*) reporter gene construct pYSn1 to search for clones with altered *GPXH *response [21]. The wild-type strain 4A^+ ^transformed with pYSn1 was UV-mutagenized and colonies were grown on TAP plates in the dark. Then, a total number of 5500 clones were analyzed for their *GPXH-ARS *expression under medium light (ML) condition of 80 μmol photons m^-2 ^s^-1 ^in the presence or absence of 1 μM NR. Average *GPXH*-*ARS *expression was induced 6.0 ± 2.0 fold by NR treatment. A cutoff of 2.5-fold induction by NR and 2.2 fold higher expression was used to select for clones with reduced induction or increased basal expression, resulting in 22 *GPXH-ARS *induction deficient (*gid*) and 41 *GPXH-ARS *overexpressor (*gox*) mutants (Figure 1A). However, after retesting these clones, only six *gid *and 32 *gox *mutants could be confirmed.

Altered response of the reporter enzyme in 4A^+ ^pYSn1 can result from mutations affecting the cellular ARS activity, the production of ^1^O_2 _or the signal transduction of ^1^O_2_-induced gene expression. To identify the first class of mutants, the induction of the endogenous *GPXH *gene was measured in the six *gid *mutants exposed to NR. Unfortunately, all *gid *mutants retained full induction of the *GPXH *wild-type gene (data not shown). For the 32 *gox *mutants, a secondary screen was performed to reduce the number of strains for *GPXH *expression analysis. Based on the knowledge that *GPXH *overexpression increases the resistance of *C. reinhardtii *to chemicals enhancing ROS production [25], the mutants were exposed to the ^1^O_2_-producing photosensitizers RB and NR at ML conditions or the O_2_^.-^-producing chemicals metronidazole (MZ) and methyl viologen (MV) and the organic tert-butylhydroperoxide (t-BOOH) under low light intensity (LL, 15 μmol photons m^-2 ^s^-1^). One group of mutants with 13 members was more resistant to t-BOOH compared to wild-type, and many but not all of these mutants were also resistant to NR and RB (Table 1). Furthermore, all strains were tested for their tolerance to high light intensities (HL, 500 μmol photons m^-2 ^s^-1^) resulting in the identification of 11 HL-sensitive clones. This phenotype was often combined with sensitivity to RB, NR, MZ or MV. A third group of mutants (12 clones) showed no or only very weak changes in tolerance to oxidative stress. Since for this subset of mutants a relatively low *GPXH-ARS *overexpression was determined, they were excluded from further analysis.

**Table 1:** Tolerance of gox mutants to various oxidative stress conditions

Mutant	HL	t-BOOH	NR	RB	MZ	MV
22D2	S	n	n	n	S	s
22D1	S	n	n	s	S	n
21E2	S	n	n	n	S	n
15B10	S	n	n	n	S	n
18C2	S	n	n	s	n	n
18F6	S	n	n	n	n	n
14H8	S	n	n	n	r	n
18G9	S	R	r	n	r	R
21B4	S	R	S	n	S	n
26D5	S	r	n	n	n	n
14A9	s	r	n	r	n	n
14B5	n	R	r	r	n	r
14C11	n	r	r	r	n	n
15H8	n	R	R	r	n	n
35H11	n	R	R	r	n	n
20H4	n	R	r	r	n	n
18B11	n	r	R	n	n	n
13D3	n	R	n	n	n	n
13H11	n	R	n	s	n	n
19H4	n	R	s	s	S	r

Abbreviations: HL: high light of 500 μmol photons m^-2 ^s^-1 ^PAR, t-BOOH: tert-butylhydroperoxide, NR: neutral red, RB: rose bengal, MZ: metronidazole, MV: methyl viologen. Tolerance was classified in five different categories compared to the wild-type strain: **S: **very sensitive, **s: **sensitive, **n: **no difference from wild-type, **r: **resistant, **R: **very resistant. Mutants could be divided into three different groups: HL sensitive mutants, t-BOOH resistant mutants and mutants with no or only minor changes in tolerance (not shown).

The remaining 20 mutants, being either HL-sensitive and/or t-BOOH resistant, were then tested for the expression of the endogenous *GPXH *wild-type gene by qPCR. A significantly (P < 0.05) stimulated expression compared to the corresponding wild-type strain could be detected in seven clones ranging from 1.4- to 5.5-fold overexpression (Additional file 1). Even though there was a clear correlation (R^2 ^= 0.76) between *GPXH *expression and the expression of the reporter construct, all 20 mutants had a stronger overexpression of *GPXH-ARS *measured by enzyme activity than *GPXH *expression determined by qPCR, which might be the consequence of individual mRNA or the reporter enzyme stability (Figure 1B).

**Figure 2:**
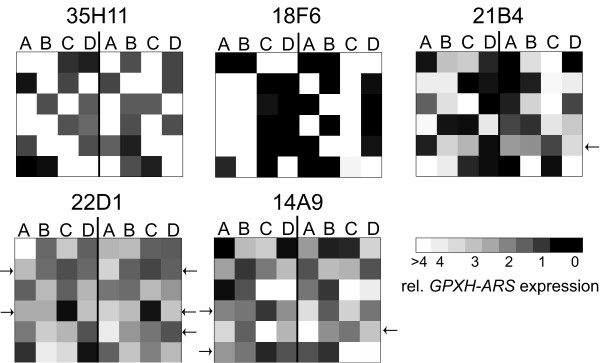
**Segregation analysis of the *GPXH-ARS *overexpression in five selected *gox *mutants**. Complete tetrads (A-D) of 12 independent backcrosses with the unmutagenized strain 4A^- ^pYS1 were tested for *GPXH-ARS *expression under control condition shown as relative expression levels using the indicated grey scale code. Tetrads where more than two progenies have similar expression levels diverge from typical 2:2 segregation expected if a single nuclear gene would be affected in the mutant. These tetrads are indicated by arrows.

High doses of UV radiation as applied in this experiment induce multiple point mutations in the genome. To analyze whether defects in multiple genes might be responsible for the phenotype, we performed tetrad analysis of selected *gox *mutants by crossing them back to the strain 4A^- ^pYS1. Twelve independent tetrads were tested for segregation of high *GPXH-ARS *expression in each mutant. A clear 2:2 segregation of the wild-type and mutant phenotypes was found in strains 35H11 and 18F6 (Figure 2), indicating that mutations in a single nuclear gene is responsible for the increased *GPXH *expression in each case. The same was true for 21B4 except for one tetrad where one high, two medium and one low expressing progeny were found, suggesting that two closely linked mutations might be responsible for the high ARS activity phenotype. No consistent 2:2 segregation was found in backcrosses of strain 22D1 and 14A9. Whereas for 22D1 at least six tetrads resulted in either 3:1 or 1:2:1 segregations, for 14A9 the pattern of three tetrads differed from standard single allele segregation.

### *GPXH *overexpression in *gox *mutants due to increased singlet oxygen production

**Figure 3:**
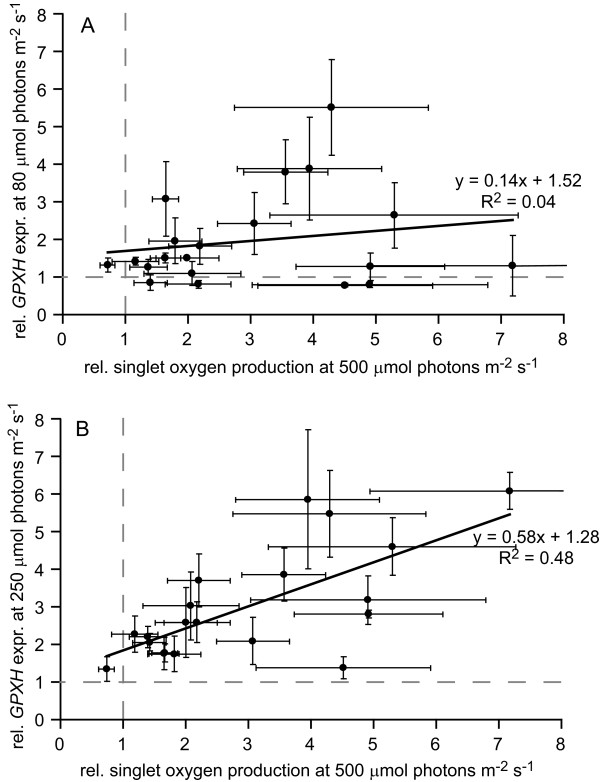
**Correlation of ^1^O_2 _production and *GPXH *expression**. Singlet oxygen production was measured with SOSG in each of the 20 *gox *mutants during short-term exposure to HL (500 μmol photons m^-2 ^s^-1 ^for 15 min), and was plotted against *GPXH *expression of the corresponding mutant grown either under (**A**) ML- (80 μmol photons m^-2 ^s^-1^) or (**B**) HL-condition (250 μmol photons m^-2 ^s^-1^). This revealed that ^1^O_2 _production in the mutants positively correlates with *GPXH *expression under HL- (R^2 ^= 0.48) but not ML-conditions (R^2 ^= 0.04). The production of ^1^O_2 _was calculated for each mutant from five and *GPXH *expression from three independent experiments (average ± SE), and normalized to the corresponding level of the wild-type strain (grey dashed lines).

Stimulated *GPXH *expression might either be due to a stimulated production of ^1^O_2 _or a constitutively active signaling pathway under LL condition. Sensitivity to HL intensity of several mutants indicates that the former might be the reason for high *GPXH *expression in some of the *gox *mutants. The formation of ^1^O_2 _was therefore measured with the fluorescent dye singlet oxygen sensor green (SOSG) allowing the specific quantification of ^1^O_2 _[26]. In order to detect significant amounts of ^1^O_2 _with the membrane impermeable SOSG in the wild-type strain, the cells had to be broken by freezing, and exposed to HL intensity (500 μmol photons m^-2 ^s^-1^) for a period of 15 min. Increased ^1^O_2 _formation compared to wild-type (at least 1.8 fold) was detected in all but one of the HL-sensitive mutants (Figure 3 Additional file 2). Surprisingly, several mutants with normal resistance to HL also showed a stimulated ^1^O_2 _production even though the difference to wild-type was not always significant (P < 0.05).

Comparing ^1^O_2 _formation with *GPXH *overexpression in *gox *mutants, no direct correlation (R^2 ^= 0.04) between the two parameters could be found (Figure 3A). However, since the formation of ^1^O_2 _is a light intensity-dependent photoreaction and *GPXH *expression and ^1^O_2 _formation were quantified at different light intensities (80 and 500 μmol photons m^-2 ^s^-1^), it is difficult to directly compare these parameters. We therefore measured *GPXH *expression in all 20 *gox *mutants at the highest possible light intensity at which the mutants could still survive for at least 24 h (250 μmol photons m^-2 ^s^-1^) and which thus corresponds to HL conditions. Indeed, a much stronger stimulation of *GPXH *expression compared to wild-type could be detected in more than half of the mutants, resulting in a stronger correlation (R^2 ^= 0.48) between ^1^O_2 _formation and *GPXH *expression (Figure 3BAdditional file 1).

**Figure 4:**
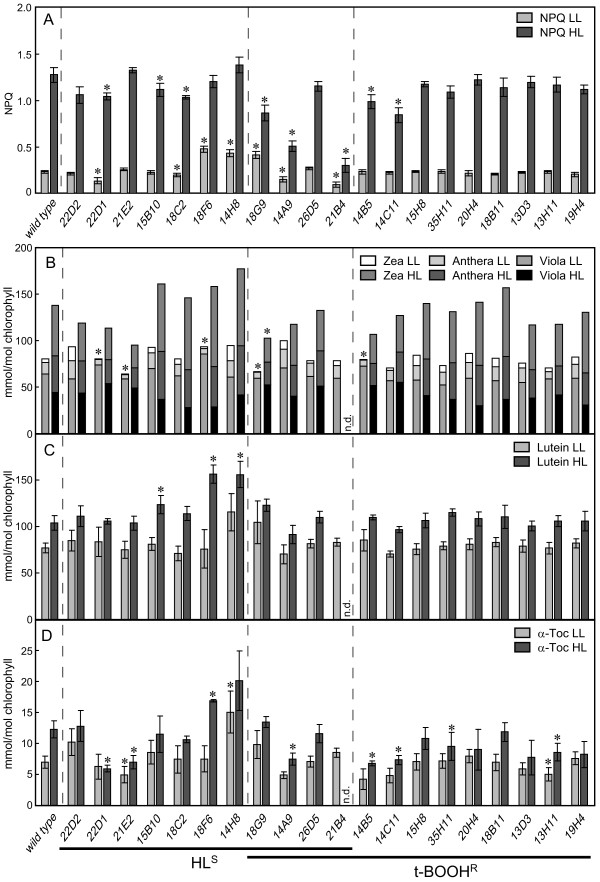
**Carotenoid content and NPQ in the isolated *gox *mutants**. Non-photochemical quenching of chlorophyll fluorescence (NPQ) (**A**), carotenoid contents (**B-C**), and α-tocopherol content (**D**) of 20 *gox *mutants were analyzed in cultures grown either under LL (15 μmol photons m^-2 ^s^-1^) or HL conditions (250 μmol photons m^-2 ^s^-1^). The order of clones is the same as in Table 1, divided into HL sensitive and t-BOOH resistant mutants (n.d.: not determined). Data show averages from 4-5 independent experiments (± SE), and significant (P < 0.05) differences from wild-type are indicated by a star (in **B **significance of deepoxidation of xanthophylls ([Z+A]/[Z+A+V]) is shown).

Increased levels of ^1^O_2 _in *gox *mutants are a consequence of either increased generation or lowered detoxification of ^1^O_2_. The former might result from a deficient photoprotection mechanism, such as a reduced capacity of NPQ. Therefore, NPQ was measured in the *gox *mutants acclimated to either LL (15 μmol photons m^-2 ^s^-1^) or HL (250 μmol photons m^-2 ^s^-1^) for at least 24 h. Four mutants (22D1, 18C2, 14A9 and 21B4) had significantly reduced NPQ under both LL and HL conditions (Figure 4A). Four other strains (15B10, 18G9, 14B5 and 14C11) only had lower NPQ under HL but not LL conditions, suggesting a light intensity dependent effect, and for three mutants (18F6, 14H8 and 18G9) a stimulation of NPQ even at LL conditions was found.

**Figure 5:**
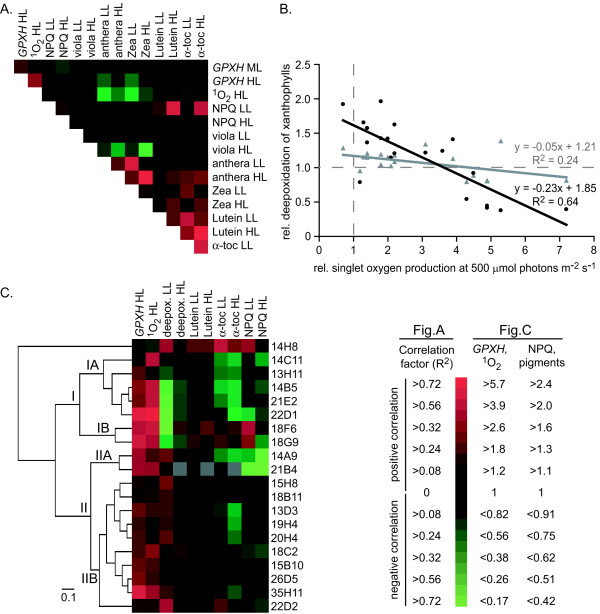
**Correlation of various analyzed parameters in the isolated *gox *mutants**. **A**. The linear correlation coefficient R^2 ^between each combination of the parameter tested in the 20 *gox *mutants was calculated and values were translated into a color code according the scale indicated. **B**. The correlation between ^1^O_2 _production at HL and deepoxidation of xanthophylls ([Z+A]/[Z+A+V]) at LL (black circles) and HL (grey triangles) is shown. **C**. Cluster analysis of 20 *gox *mutants based on *GPXH *expression, ^1^O_2 _production, NPQ and pigment contents under either LL or HL condition as shown in Figure 3 and 4. All data are relative to wild-type levels under the same growth condition and shown in a color code according the scale indicated. The nature of different groups of mutants is discussed in the text.

Energy-dependent quenching (qE) is one component of NPQ that requires synthesis of the xanthophylls, zeaxathin and antheraxanthin. However, these and other carotenoids as well as α-tocopherol are also important antioxidants involved in detoxification of ^1^O_2_. Pigment analysis of the 20 *gox *mutants acclimated to the same LL or HL intensity as for NPQ measurements revealed only small changes in pigment contents of few strains. Lutein was not altered in LL and only slightly higher in three mutants in HL conditions compared to the wild-type strain (Figure 4C). Similarly, α-tocopherol was not strongly affected in LL condition, except for 14H8, but was significantly reduced in 7 mutants after exposure to HL for 24 h (Figure 4D). For only one strain, 18F6, higher levels of α-tocopherol than in wild-type were detected under HL conditions, and this correlated with increased lutein and zeaxanthin levels in this mutant. Despite the important role of zeaxanthin and antheraxanthin in photoprotection and prevention of ^1^O_2 _generation, only moderate changes of these xanthophylls were detected in the mutants compared to the wild-type. Only one mutant (18G9) had a significantly reduced de-epoxidation of xanthophylls during HL exposure showing that this process seems still to be functional in all mutants (Figure 4B). Nevertheless, when grown in LL, five mutants had significantly reduced de-epoxidation states whereas other mutants had rather increased levels of zeaxanthin and antheraxanthin (Figure 4B and 5B).

### *GPXH *expression and singlet oxygen production negatively correlate with the xanthophyll de-epoxidation state

In order to analyze the relationship of all the parameters measured in the 20 *gox *mutants, linear correlation factors were calculated for every possible combination of parameters (Figure 5A). Not surprisingly, a strong positive correlation between antheraxanthin and zeaxanthin levels was found, which negatively correlated with violaxanthin under HL conditions, as expected from operation of the xanthophyll cycle. As already shown in Figure 3B, ^1^O_2 _ formation correlated with *GPXH *expression under HL conditions. Both parameters also negatively correlated with antheraxanthin and zeaxanthin levels, especially in LL-grown cultures. Thus, when comparing ^1^O_2 _production at HL and xanthophyll levels at LL it was striking that all the mutants but one (14C11) had a high de-epoxidation state of xanthophylls or increased ^1^O_2 _production compared to wild-type (Figure 5B). This effect was less pronounced when ^1^O_2 _production was compared with de-epoxidation at HL because strongly stimulated de-epoxidation reduced the relative differences between the clones. Finally, antheraxanthin and zeaxanthin levels of HL-grown cultures weakly correlated with α-tocopherol and lutein contents under this light condition.

Hierachical clustering of the mutants with all measured parameters was performed to test whether there are groups of mutants with similar phenotypic pattern. These analyses revealed that one mutant (14H8) behaved differently from all other mutants (Figure 5C). Even though 14H8 was originally screened for high *GPXH*-*ARS *expression and was found to be HL sensitive, it did not show any stimulated *GPXH *expression or ^1^O_2 _production at HL intensities. All other mutants were divided into two major groups (I and II) mainly based on the de-epoxidation state of the xanthophyll pool. Group I had lower levels of antheraxanthin and zeaxanthin at LL than wild-type and most of these mutants showed high ^1^O_2 _production and increased *GPXH *expression. They could be further subdivided into two groups, where strain 18F6 and 18G9 belonged to one group (IB) with stimulated NPQ in LL conditions and no changes in α-tocopherol levels under any light intensity. Mutants of the other subgroup (IA), on the other hand, had strongly reduced α-tocopherol levels, especially in HL grown cultures, and either lowered or not changed NPQ compared to the wild-type strain.

The second group of mutants (II) with similar or slightly increased xanthophyll levels to wild-type had generally a much lower stimulation of ^1^O_2 _production and *GPXH *expression than mutants of group I. Exceptions were clones 14A9 and 21B4 from subgroup IIA with strong *GPXH *overexpression and stimulated ^1^O_2 _production, but these mutants showed strongly reduced NPQ under both LL and HL intensity that was probably responsible for these phenotypes. In the other subgroup (IIB) only two mutants (35H11 and 15B10) had significant *GPXH *overexpression and higher ^1^O_2 _production and except for lower α-tocopherol levels in HL for some mutants, little differences compared to wild-type were detected for most parameters.

## Discussion

### No mutants with deficient *GPXH *induction

Screening for mutants with altered *GPXH *expression resulted in the isolation of several high *GPXH *expression mutants but no strain with deficient or strongly reduced *GPXH *induction by ^1^O_2 _could be isolated. With a total number of 5500 UV-mutagenized clones tested we assume that the coverage of mutations in the genome should be high enough to hit at least one component of a putative ^1^O_2 _signaling cascade with a loss-of-function mutation. The relatively high UV dose resulting in the survival of only 0.4 to 2% of the cells and the fact that during segregation analyses at least two out of five *gox *mutants contained two mutations affecting the phenotype suggested a high mutation density in the screened population. Still, a second mutant screen for suppressor mutants of *GPXH *overexpression in strain 21B4 was performed using an additional plasmid containing the *GPXH *promoter in front of the nitrate reductase gene (data not shown). This enabled a direct selection for reduced expression under low light conditions by selecting for chlorate-sensitive clones. Out of a total number of 2 × 10^6 ^cells surviving UV mutagenesis, 1060 clones were chlorate resistant, and of those, 15 clones also had low induction (< 2 fold) of the *GPXH*-*ARS *reporter construct by NR. However, all of these clones showed normal induction of the wild-type *GPXH *gene, indicating that under these screening conditions no *gid *mutants can be isolated. The conclusion that in strain 21B4 a constant increased ^1^O_2 _production is responsible for high *GPXH *expression might explain why no *GPXH *induction deficient mutants could be isolated, because *GPXH *might be essential for defense against ^1^O_2_-induced damage. This can be excluded for the original mutant screen because selection against light-sensitive mutants during the recovery phase after mutagenesis was prevented by growing the 5500 clones in the dark. Furthermore, none of the clones was light sensitive under ML conditions during the induction tests. Thus, even though steps were taken to minimize a negative selection against *gid *mutants, we cannot exclude that a functional ^1^O_2 _response of the *GPXH *and maybe other genes is essential for *C. reinhardtii*. On the other hand, it is also possible that several redundant signaling pathways form a complex network to activate *GPXH *expression, thereby hampering the isolation of *gid *mutants. Finally, it still might be that more dark-grown mutagenized clones had to be tested to find the desired mutants. A similar screen to isolate ^1^O_2 _responsive mutants in *A. thaliana *resulted in the identification of at least three mutants deficient in the upregulation of different ^1^O_2_-induced genes in the conditional *flu *mutant [27]. However, ^1^O_2_-signaling seems to have different cellular functions in *A. thaliana*, at least in seedlings, where it is part of a programmed cell death response and in *C. reinhardtii *where it seems to be involved in response to cytotoxic environmental stresses [20,28].

### *GPXH *overexpression and correlation with singlet oxygen production, NPQ and pigment levels

In contrast to the lack of *gid *mutants, many *gox *mutants with a stimulated expression of the *GPXH *wild-type gene, especially under HL intensities, could be isolated (Figure 3). The light intensity-dependent increase of *GPXH *overexpression and the sensitivity to HL conditions indicated that in several mutants ^1^O_2 _formation might be enhanced and cause a photooxidative stress. Indeed, increased ^1^O_2 _formation was measured in all but one HL-sensitive as well as many HL-resistant *gox *mutants (Additional file 2), which nicely correlated with *GPXH *expression under HL conditions (Figure 3B). This indicates that in most or even all *gox *mutants, *GPXH *overexpression seems not to be caused by a constitutively active signal transduction pathway but by the increased production of ^1^O_2 _under the light conditions tested. The poor correlation between ^1^O_2 _production and *GPXH *expression under ML (Figure 3A), the clustering in at least five phenotypic distinct groups (Figure 5C) and the fact that three of the mutants (18F6, 21B4 and 35H11) have been mapped to different linkage groups (data not shown) indicates that mutations in different nuclear genes are responsible for the increased ^1^O_2 _production in the various mutants.

Singlet oxygen formation in photosynthetic organisms is caused by the conversion of excited chlorophylls into the triplet state and the reaction with molecular oxygen [3]. The organisms try to minimize this process by regulating the excitation pressure on the PSII reaction center chlorophylls, optimizing the electron flow in the photosynthetic electron transport chain and quenching chlorophyll triplet states and ^1^O_2_. Thus, ^1^O_2 _accumulation can result from either stimulated production, e.g. due to enhanced triplet chlorophyll formation, or lowered detoxification of the ROS due to defects in some light-induced protection mechanisms. Such a defect in protection mechanisms could be the reason for high ^1^O_2 _formation in most of the group I mutants of cluster analysis (Figure 5C) having reduced zeaxanthin and antheraxanthin levels which correlates with high ^1^O_2 _formation (Figure 5B). A well characterized mutants with reduced xanthophyll levels and defects in photoprotection is the *npq1 lor1 *double mutant lacking the carotenoids zeaxanthin, antheraxanthin and lutein. Similar to many mutants of group I, this mutant exhibits increased ROS production, *GPXH *expression and sensitivity upon HL but not LL illumination [16,29]. However, comparison of *npq1 *and *npq1 lor1 *showed that reduced levels of zeaxanthin and antheraxanthin but normal levels of other carotenoids like lutein would probably not cause a very strong phenotype [16,30]. Thus, reduced efficiency of more than one protection mechanism might be required to achieve increased ^1^O_2 _accumulation. Indeed, five of the group I mutants with reduced xanthophyll levels (13H11, 14B5, 14C11, 21E2, 22D1) also showed lowered α-tocopherol levels under LL and/or HL conditions (Figure 5C, group IA). Furthermore, levels of other components of the thylakoid membrane with ^1^O_2_-quenching capacity like plastoquinone and α-tocopherolquinone [31,32] were not quantified but might also be affected in some of the mutants. Thus, reduced photoprotection by the combination of lower xanthophylls, α-tocopherol and maybe other deficiencies might be the cause for increased ^1^O_2 _production and *GPXH *expression in these mutants. This could either result from mutations in multiple loci, as found for clone 22D1 (Figure 3), or caused by mutations in a single gene encoding for a global regulator of cellular photoprotection mechanisms.

Even though deepoxidation of xanthophylls is involved in NPQ [33], these parameters did not correlate in our mutants (R^2 ^< 0.01) indicating that other factors also play an important role for NPQ. For example, two strains of group I (18F6 and 18G9) showed rather increased NPQ levels under LL conditions even though their xanthophyll levels were reduced, clustering them in a separate subgroup (IB) of group I (Figure 5C). Very high *GPXH *expression in 18F6 and 18G9 under ML conditions indicates stimulated ^1^O_2 _generation already at LL. We speculate that these mutants might have increased ^1^O_2 _production due to an enhanced energy transfer from the PSII reaction center to molecular oxygen. By this, quenching of excitation energy by molecular oxygen would increase NPQ but reduce the photosynthetic electron transport rate required for building up the proton gradient and activating the xanthophyll cycle. Thus, reduced xanthophylls would not be the cause but the consequence of increased ^1^O_2 _production. However, other effects of the mutations cannot be excluded which might also explain these phenotypes.

Contrary to 18F6 and 18G9, two mutants (14A9 and 21B4 in group IIA) had strongly reduced NPQ both under LL and HL conditions but similar or rather increased xanthophyll levels compared to wild-type showing that defects in qE-independent mechanisms seem to affect NPQ in these mutants. Photoinhibition (qI) stimulated by excess light should not be relevant under LL conditions, and a deficiency in state transition (qT) should not strongly affect NPQ at HL intensities. On the other hand, a qE-independent effect was also found in a *C. reinhardtii *mutant defective in two linked *LHCSR3 *genes [10]. However, segregation analysis of backcrosses of 14A9 and 21B4 with a wild-type strain revealed that both mutants have probably mutations in two different genes affecting *GPXH *expression (Figure 2) indicating that the phenotypes of these mutants could be caused by the combination of different defects.

In mutants with functional detoxification mechanisms, strong ^1^O_2 _accumulation after stimulated production of the ROS might be prevented by the induction of these detoxification mechanisms. This is supported by the negative correlation of ^1^O_2 _formation and deepoxidation of xanthophylls, where increased levels of zeaxanthin and antheraxanthin correlate with a low stimulation of ^1^O_2 _production (Figure 5B). Mutants with increased xanthophyll levels are mainly represented in the group IIB mutants of cluster analysis with rather few and weak phenotypic changes (Figure 5C). A general increase in antioxidant levels including zeaxanthin, antheraxanthin, lutein and α-tocopherol and a significant rise in NPQ was detected in strain 14H8, where no stimulated ^1^O_2 _production and *GPXH *expression could be measured any more. This shows that the various protection mechanisms can compensate each other and thus control the production of deleterious ROS. This is in agreement with data of various mutants lacking specific antioxidants: α-tocopherol-deficient strains of *C. reinhardtii*, *Synechocystis sp*. PCC6803 and *A. thaliana *all were very tolerant to photooxidative stress during HL conditions, and only under extreme conditions such as a combination of very HL and low temperature or chemical treatment a phenotype became visible [34-37]. It was suggested that the presence of other antioxidants such as zeaxanthin or increased levels of β-tocopherol can compensate for α-tocopherol deficiency [35,37]. Conversely, the *A. thaliana npq1 *mutant, lacking zeaxanthin and antheraxanthin, accumulates higher amounts of α-tocopherol [38]. Thus, zeaxanthin, α-tocopherol and plastoquinol have overlapping functions in photoprotection and together prevent the formation of deleterious ^1^O_2 _under natural conditions [8,32,39-42]. However, when these protection mechanisms are overwhelmed, ^1^O_2 _starts to accumulate and damage cellular components. This is when defense genes like *GPXH*, which repair and remove damaged biomolecules, are required to survive the oxidative stress. Activation of genetic stress response without altering antioxidant levels by an acclimation to increased ^1^O_2 _production or the direct overexpression of the *GPXH *gene in *C. reinhardtii *were shown to increased resistance to oxidative stress by RB, NR and t-BOOH [20,25]. Increased tolerance against t-BOOH was also found for 13 of the 32 *gox *mutants tested including all the group IIB mutants as well as strains 14B5, 14C11 and 35H11 (Table 1). Thus, increased expression of stress response genes like *GPXH *might explain the HL resistance of these mutants and shows the important role of *GPXH *and other defense genes in the photooxidative stress response of photosynthetic organisms.

## Conclusions

The failure to isolate ^1^O_2 _signal transduction mutants indicates that several redundant signaling pathways might be involved in the *GPXH *response. This is supported by the identification of multiple regulatory elements in the *GPXH *promoter being required for induction of the gene [21]. Singlet oxygen generation, on the other hand, was altered in several mutants resulting in higher expression of the *GPXH *gene. Increased oxidative stress resistance of many of these mutants confirms the importance of the ^1^O_2_-induced genetic response in the defense against ROS-induced damage. Furthermore, isolation of phenotypic different groups of ^1^O_2_-overproducing mutants indicates that mutations in different photoprotective mechanisms might be responsible for higher ^1^O_2 _levels in various *gox *mutants which most seem to be, based on pigment analysis, different from known photoprotective mutants like *npq1 lor1*. The comparison of their phenotypes suggests that in several *gox *mutants multiple defense processes might be affected what might be due to, among other things, mutations in a global regulator of cellular photoprotection mechanisms. Thus, the isolation of these mutants might allow identifying new components involved in the control of ^1^O_2 _formation by different cellular protection mechanisms.

## Methods

### Strains and growth conditions

The *C. reinhardtii *strain used to generate the population of mutants was 4A^+ ^pYSn1, which is in a 137c (CC-125) background [43]. This strain was generated by co-transformation of 4A^+ ^with the plasmids pYSn1 containing the *GPXH*-arylsulfatase reporter construct [21] and pBC1 containing the *Streptomyces *aminoglycoside 3'-phosphotransferase typeVIII encoding gene (*aphVIII*) for selection of transformants on paromomycin [44]. A near-isogenic mt^- ^strain (4A^- ^pYSn1) was obtained by crossing 4A^+ ^pYS1 with 4A^- ^[43] and used to backcross mutants for segregation analysis.

All strains were grown heterotrophically in Tris-Acetate-Phosphate-medium (TAP) [45] either on 1.5% agar plates or in liquid cultures agitated on a rotary shaker (120 rpm) at 22°C and the light conditions indicated. For storage, mutants were kept on TAP agar plates in dim light.

### Screening for *GPXH *expression mutants

UV mutagenesis was performed in an UV Stratalinker™1800 (Stratagene, CA). Cells were grown heterotrophically to a density of 5 × 10^6 ^cells ml^-1^, and 20 ml were aliquoted into a sterile Pyrex^® ^petri dish (14 cm diameter) and exposed to 30 - 60 mJ cm^-1 ^of UV light. Cells were kept in the dark for 1 day immediately following UV treatment to prevent initiation of light-activated DNA repair mechanisms. Then the UV-mutagenized cells were spread on TAP plates and incubated in the dark until colonies appeared.

A total of 5500 colonies were picked, transferred onto fresh TAP plates and maintained at low light (LL, 15 μmol photons m^-2 ^s^-1^) for 5 to 10 days. These clones were used to inoculate 150 μl TAP in 96-well plates by replica plating. After 2 days of growth at medium light (ML, 80 μmol photons m^-2 ^s^-1^), another 150 μl of TAP was added to the cultures, mixed and divided into two separate 96-well plates. To induce *GPXH *expression, NR (1 μM) was added to one of the plates and incubated at ML for 8 hours. Arylsulfatase activity was analyzed by adding 7.5 μl of a 20 × GIN solution (1 M glycine-NaOH pH 9.0, 0.4 M imidazole, and 180 mM *p*-nitrophenylsulfate) and measuring absorbance at 410 nm after 0, 5, 10 and 20 min of incubation at 35°C. In parallel, absorbance at 750 nm was measured to determine cell density and normalized ARS activity was calculated with the following equation:

$$\text{ARS}\;\text{activity}=(\text{slope}({\text{OD}}_{410}-{\text{OD}}_{750};\text{t}))/{\text{OD}}_{750}$$

Relative *GPXH-ARS *expression was then calculated for each clone by dividing its ARS activity by the average control ARS activity. After the initial selection for altered *ARS *expression, the clones were rescreened three more times under the same exposure conditions to ensure reproducible changes in the selected mutants.

### Testing for resistance phenotypes

Resistance to different oxidative stress conditions was tested by inoculating the clones in 150 μl TAP in a 96-well plate and incubation at ML for 2 days. Then 5 μl of each cultures was spotted on TAP plates containing the following chemicals: tert-butylhydroperoxide (t-BOOH: 100, 150, 200 and 250 μM), neutral red (NR: 4, 5, 8, 12 and 18 μM), rose bengal (RB: 2, 2.5, 3, 4 and 5 μM), metronidazole (MZ: 1, 2, 3, 5 and 8 mM), methyl viologen (MV: 0.5, 1, 1.5, 2 and 3 μM). The plates were incubated at either LL (t-BOOH, MZ and MV) or ML (NR and RB), together with a control plate without any chemical for 3 to 4 days depending on the light intensity. High light resistance was tested by exposing 5 μl of cultures on a TAP plate to 500 μmol photons m^-2 ^s^-1 ^(HL) for 3 days.

### Segregation analysis

Six mutants (14A9, 18F6, 18G9, 21B4, 22D1 and 35H11) were analyzed genetically for segregation of their phenotypes by crossing them to strain 4A^- ^pYS1. The resulting zygospores were harvested and dissected as described in Harris (1989). For each mutant a total of twelve tetrads with four surviving cells each were analyzed for their *GPXH-ARS *expression pattern except for 18G9, which did not result in any viable progenies. *GPXH-ARS *expression analysis was performed as described above in three replicates per clone.

### Singlet oxygen formation

Cultures grown under LL to 3 × 10^6 ^cells ml^-1 ^were adjusted to 3 μg ml^-1 ^chlorophyll content, and 0.5 ml samples were frozen in liquid nitrogen to lyse the cells. After thawing, 100 μl of cell suspension were transferred to a black 96-well plate. To all the samples including two medium-only controls for background correction, 1 μl of a 1 mM Singlet Oxygen Sensor Green (SOSG) (Invitrogen/Molecular Probes) solution was added. Fluorescence levels were measured as peak height at 530 nm (excitation 480 nm) [26] after 0 and 15 min of exposure to HL conditions (500 μmol photons m^-2 ^s^-1^) in a Tecan infinite^® ^200 fluorescence plate reader. To confirm ^1^O_2 _detection by SOSG, fluorescence spectra of cultures exposed to the same light conditions in the presence of either 10 mM of the ^1^O_2 _quencher 1,4-diazabicyclo[2.2.2]octane (DABCO) or in medium containing 50% deuterium oxide (D_2_O) were measured representatively for strain 22D1 in three independent replicates (Additional file 3). As expected, DABCO reduced the fluorescence signal of SOSG by quenching ^1^O_2 _whereas D_2_O stimulated the signal because D_2_O increases the ^1^O_2 _lifetime. Furthermore, no increased SOSG fluorescence could be measured in dark incubated algal samples compared to medium-only controls (background) confirming the detection of ^1^O_2 _by SOSG. Singlet oxygen production for each strain was determined by calculating the slope of the increase in background-corrected SOSG fluorescent signals during the 15 min of exposure, and relative values were calculated for each mutant by dividing its levels by the levels of ^1^O_2 _production in the wild-type strain.

### RNA isolation and quantitative real-time PCR (qPCR)

Cells of 5 ml cultures grown to 3 × 10^6 ^cells ml^-1 ^at the light condition indicated were harvested by centrifugation. Total RNA was isolated by the Trizol method as described earlier [46]. For qPCR experiments, 200 ng of individual total RNA was used in each 10 μl reverse transcription reaction with a reverse transcription kit (Applied Biosystems) according to the manufacturer's instruction.

Sequences of primers for qPCR were designed with the Primer Express™software (Applied Biosystems). qPCR reactions were performed on the ABI Prism^® ^7000 or 7500 Sequence Detection System (Applied Biosystems) as described earlier [46]. Threshold cycle (C_t_) values were determined for all reactions in the logarithmic amplification phase, and the average C_t _value was calculated for each sample out of three technical replicates. Each reaction was confirmed to contain a single amplicon by gel electrophoresis and melting curves. Genomic DNA contamination in RNA samples was assessed by qPCR using RNA without reverse transcription as templates, and in all cases had at least six C_t_-values higher than that of respective cDNAs. The efficiency for the amplification of each product was determined by serial dilutions of template cDNA and used to correct C_t _values for variable amplification efficiencies. The C_t _values of the *CBLP *gene were used as the internal references. Relative expression was calculated for each mutant compared to 4A^+ ^pYS1 under the same growth conditions as an average with standard error out of three independent experiments.

### Pigment analyses

For tocopherol and pigment analysis, cells from 1 ml samples were harvested by centrifugation and immediately frozen in liquid nitrogen. Pigments and α-tocopherol were extracted by vortexing in 300 μl of acetone for 1 min, and the extracts were filtered through 2 μm nylon filters. Pigments were fractionated and analyzed by high-performance liquid chromatography (HPLC) as described previously [42].

### Determination of NPQ

Chlorophyll fluorescence was determined with an imaging pulse amplitude modulated (IPAM) chlorophyll fluorescence system (IMAG-MAX/l, Walz, Germany). Mutants were grown under LL (15 μmol photons m^-2 ^s^-1^) or 250 μmol photons m^-2 ^s^-1 ^for 2 days up to a density of 3 × 10^6 ^cells ml^-1 ^before the cells of 200 μl of culture were collected on a glass microfiber filter (Whatman, UK) by vacuum filtration. To keep filters moist they were transferred on a wet paper towel and cells were dark adapted for 20 min before maximal chlorophyll fluorescence level *F_m _*was determined. Then actinic light of the same intensity as growth light was turned on for 15 min before *F_m_' *was measured to calculate average NPQ as (*F_m_-F_m_'*)/*F_m_' *of five independent replicates.

### Cluster analysis

Hierarchical gene clustering was performed using the Cluster 3.0 software. Data were first log transformed and normalized by multiplying all values of a parameter with a constant scale factor so that the sum of the squares of the values for each parameter was 1.0. This resulted in a scale factor for *GPXH *expression and ^1^O_2 _formation of 7.1. For NPQ and pigment contents, which had similar maximal levels, a single scale factor (3.5) was calculated based on the parameter with the highest sum of squares (deepoxidation at LL). Clustering was performed based on average linkage and the results were visualized in a color-based expression pattern using the TreeView 1.60 software designed by the Eisen Lab http://rana.lbl.gov/.

### Statistical analysis

Different parameters determined for individual mutant were analyzed for their significant differences to the same parameters measured in the wild-type strain using a paired Student's *t*-test. Significant differences at a *p*-value < 0.05 are indicated by a star. Correlations of different parameters were analyzed by linear regression and indicated by the square of the corresponding regression coefficient (R^2^).

## Authors' contributions

BBF designed and performed the experiments, evaluated the data and drafted the manuscript. RILE and KKN contributed to conception and design of the experiments, data interpretation and drafting the manuscript. All authors have read and approved the final manuscript.

## Supplementary Material

Additional file 1 ***GPXH-ARS *and *GPXH *expression**. Click here for file

Additional file 2 **Sensitivity and relative ^1^O_2 _formation of the various *gox *mutants**. Click here for file

Additional file 3 **Fluorescence spectra of SOSG**. The fluorescence spectra were monitored representatively in samples of strain 22D1 exposed to HL-conditions for 15 min (grey lines) in either normal TAP medium (full line), TAP with 10 mM of the ^1^O_2 _quencher 1,4-diazabicyclo[2.2.2]octane (DABCO) (dashed line) or medium containing 50% deuterium oxide (D_2_O) (dash-dotted line) which increases the lifetime of ^1^O_2_. As control, dark incubated samples (black lines) in the presence (full line) or absence of algae (dotted line) (SOSG background) are shown. Click here for file
