# Supplementary material for: Characterization of singlet oxygen-accumulating mutants isolated in a screen for altered oxidative stress response in Chlamydomonas reinhardtii
Source: BMC Plant Biol. 2010 Dec 17;10:279. doi: 10.1186/1471-2229-10-279 (PMC3022906; doi:10.1186/1471-2229-10-279)
Supplement: Additional file 1 — GPXH-ARS and GPXH expression. [file 1471-2229-10-279-S1.PDF]

gox **mutant**	***GPXH-ARS* (ML)**	***GPXH* (ML)**	***GPXH* (HL)**
18G9	13.3 ± 2.0 *	5.5 ± 1.3 *	5.5 ± 1.2 *
21B4	6.2 ± 0.4 *	3.8 ± 0.9 *	3.8 ± 0.7 *
22D2	6.0 ± 1.1 *	3.1 ± 1.0 *	1.8 ± 0.2 *
35H11	5.1 ± 0.7 *	3.9 ± 1.4 *	5.8 ± 1.8 *
13H11	4.9 ± 0.7 *	1.4 ± 0.1 *	2.3 ± 0.5 *
18B11	4.8 ± 0.4 *	2.0 ± 0.6	1.7 ± 0.5
18F6	4.1 ± 1.1 *	2.6 ± 0.9 *	4.6 ± 0.8 *
18C2	3.9 ± 1.6 *	2.4 ± 0.8	2.1 ± 0.6
20H4	3.7 ± 0.5 *	1.3 ± 0.2	2.2 ± 0.3 *
14A9	3.7 ± 0.2 *	1.8 ± 0.5	3.7 ± 0.7 *
13D3	3.5 ± 0.5 *	1.5 ± 0.1 *	2.6 ± 0.9
14C11	3.4 ± 0.5 *	0.8 ± 0.0	1.4 ± 0.3
19H4	3.4 ± 0.3 *	0.8 ± 0.2	2.0 ± 0.2 *
26D5	3.1 ± 0.3 *	1.1 ± 0.3	3.0 ± 0.9 *
14H8	2.9 ± 0.3 *	1.3 ± 0.2	1.3 ± 0.3
15H8	2.8 ± 0.3 *	1.5 ± 0.1	1.8 ± 0.4
22D1	2.8 ± 0.2 *	1.3 ± 0.8	6.1 ± 0.5 *
21E2	2.7 ± 1.1 *	1.3 ± 0.4	2.8 ± 0.1 *
15B10	2.6 ± 0.4 *	0.8 ± 0.1	2.6 ± 0.5 *
14B5	2.5 ± 0.3 *	0.8 ± 0.1	3.2 ± 0.6

Relative expression of the *GPXH* wild-type and *GPXH-ARS* reporter gene in the various *gox* mutants compared to the wild-type strain was determined under the light conditions indicated.
