# Supplementary material for: Characterization of singlet oxygen-accumulating mutants isolated in a screen for altered oxidative stress response in Chlamydomonas reinhardtii
Source: BMC Plant Biol. 2010 Dec 17;10:279. doi: 10.1186/1471-2229-10-279 (PMC3022906; doi:10.1186/1471-2229-10-279)
Supplement: Additional file 2 — Sensitivity and relative 1O2 formation of the various gox mutants. [file 1471-2229-10-279-S2.PDF]

gox mutant	HL	$^1O_2$ form.
22D2	s	1.8 ± 0.2 *
22D1	s	7.2 ± 2.2 *
21E2	s	4.9 ± 1.2 *
15B10	s	2.2 ± 0.5 *
18C2	s	3.1 ± 0.6 *
18F6	s	5.3 ± 2.0 *
14H8	s	0.7 ± 0.1
18G9	s	4.3 ± 1.5
14A9	s	2.2 ± 0.5 *
26D5	s	2.1 ± 0.8
21B4	s	3.6 ± 0.7 *
14B5	n	4.9 ± 1.9 *
14C11	n	4.5 ± 1.4 *
15H8	n	1.7 ± 0.2 *
35H11	n	3.9 ± 1.1 *
20H4	n	1.4 ± 0.3
18B11	n	1.8 ± 0.4
13D3	n	2.0 ± 0.5
13H11	n	1.2 ± 0.4
19H4	n	1.4 ± 0.3

Relative values compared to the wild-type strain analyzed at 500 µmol photons m^{-2} s^{-1} are shown (s: sensitive, n: not sensitive to HL).
