# Supplementary figures and images for: Characterization of singlet oxygen-accumulating mutants isolated in a screen for altered oxidative stress response in Chlamydomonas reinhardtii
Source: BMC Plant Biol. 2010 Dec 17;10:279. doi: 10.1186/1471-2229-10-279 (PMC3022906; doi:10.1186/1471-2229-10-279)

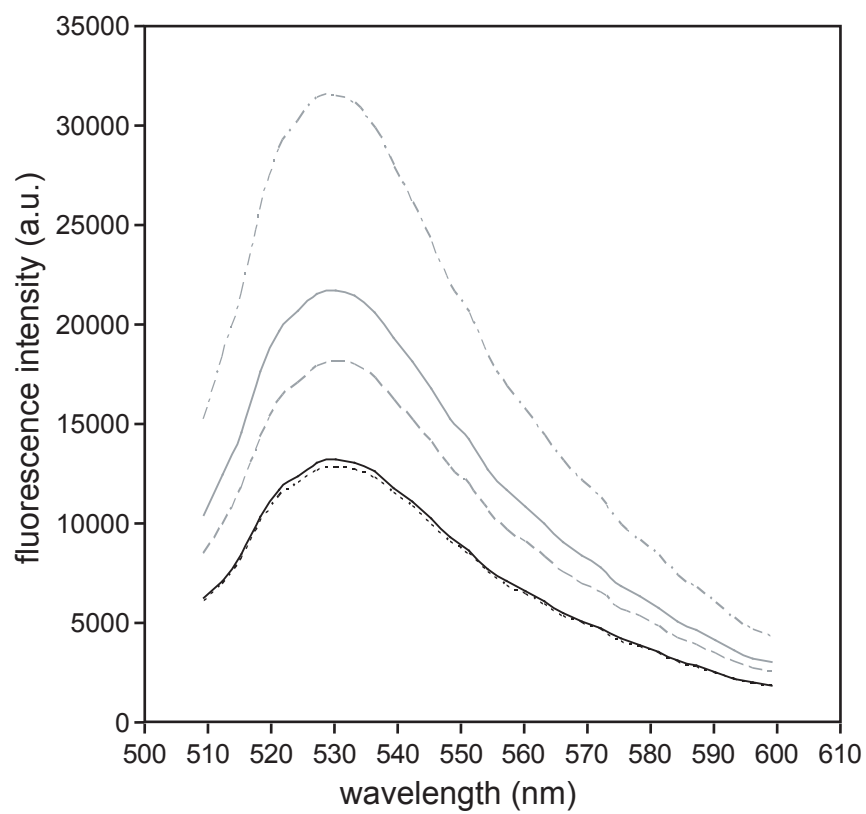

Supplement: Additional file 3 — Fluorescence spectra of SOSG. The fluorescence spectra were monitored representatively in samples of strain 22D1 exposed to HL-conditions for 15 min (grey lines) in either normal TAP medium (full line), TAP with 10 mM of the 1O2 quencher 1,4-diazabicyclo[2.2.2]octane (DABCO) (dashed line) or medium containing 50% deuterium oxide (D2O) (dash-dotted line) which increases the lifetime of 1O2. As control, dark incubated samples (black lines) in the presence (full line) or absence of algae (dotted line) (SOSG background) are shown. [file 1471-2229-10-279-S3.PDF]
